# Contrasting wood anatomy drives divergent xylogenesis and climate responses in 10 warm-temperate trees

**DOI:** 10.3389/fpls.2025.1660428

**Published:** 2026-01-16

**Authors:** Yiping Zhang, Xiaoli Zhai, Gengxin Lu, Hanwei Bai, Jiao Chen, Junliang Xu, Yaowu Tian, Neil Pederson

**Affiliations:** 1 College of Horticulture and Plant Protection, Henan University of Science and Technology, Luoyang, Henan, China; 2 Luoyang Academy of Agricultural and Forestry Sciences, Luoyang, Henan, China; 3 Independent Scholar, Maynard, MA, United States

**Keywords:** intra-annual growth pattern, leaf phenology, wood phenology, ring-porous species, diffuse-porous species, precipitation, photoperiod

## Abstract

Wood formation is crucial for understanding trees response to environmental conditions and assessing climate change impacts on carbon and water cycles. Nevertheless, species-specific differences in intra-annual radial growth dynamics remain incompletely understood. In this study, we monitored the xylogenesis, its relationships with climatic factors, and leaf phenology in 10 coexisting tree species representing contrasting wood anatomical types under warm-temperate conditions using microcoring techniques from March to December 2018. Our results showed that: (1) Semi- and ring-porous species initiated wood formation earliest (early March) and exhibited the fastest growth rates. In contrast, diffuse-porous species started xylem growth latest (late March) but completed xylogenesis earlier (early November) due to accelerated growth, while conifers maintained slower growth rates over extended growing seasons. (2) Deciduous ring-porous trees and evergreen conifers exhibited delayed leaf out relative to xylem growth, whereas diffuse-porous species initiated leaf development earlier than wood formation, highlighting differences in carbon allocation. (3) Responses to climate differed between coniferous and broadleaved trees: xylem growth of conifers correlated positively with air temperature, while broadleaved trees were primarily driven by precipitation, reflecting wood-type differences in drought sensitivity. Moreover, photoperiod effects varied among wood types, showing negative correlations for conifers and diffuse-porous species but positive correlations for (semi-)ring-porous species. These findings demonstrate distinct differences in xylogenesis between coniferous and broadleaved trees, enhancing our understanding of wood formation in angiosperms. Exploration linkages between wood formation and leaf phenology is essential for predicting species-specific responses to climate change and improving global vegetation carbon models.

## Introduction

Wood formation determines long-term carbon accumulation in trees and plays a crucial role in terrestrial carbon cycling. The process of wood formation and its sensitivity to environmental factors can reveal seasonal carbon allocation patterns, advancing our understanding of terrestrial carbon sequestration (Babst et al., 2014).

Wood formation (“xylogenesis”) encompasses developmental phases of newly formed xylem cells, including cell division, enlargement, secondary wall thickening, and maturation (Plomion et al., 2001; Wang et al., 2025b). These processes enable quantification of cell-level developmental rates, timings (onset and cessation), and durations (Li et al., 2021). Current knowledge of wood formation derives largely from studies on conifer species due to their consistent cell structure and easily identifiable tracheids (Cuny and Rathgeber, 2016; Fan et al., 2019). Eckes-Shephard et al. (2022) reviewed 17 wood formation models, 16 of which focused solely on conifers. However, xylem anatomy and the associated functional traits (leaf habitat, leaf area, wood density, etc) differ between gymnosperms and angiosperms (McCulloh et al., 2010). For example,water transport relies on tracheids in gymnosperms and vessels and tracheids in angiosperms. Advancing knowledge thus requires greater focus on broadleaved species (ring-, semi-ring-, and diffuse-porous), as current research has historically been centered on coniferous (non-porous).

In general, coniferous typically have longer growth duration but slower growth rate, while broadleaved species are able to complete wood formation faster (Martinez Del Castillo et al., 2016). Ring-porous species in temperate regions showed nearly 40% higher growth rates and 33% higher C sequestration than diffuse-porous species (Simovic et al., 2024). These differences are reflected in xylem activities timings (onset and cessation) between coexisting conifers and broadleaves (Debel et al., 2024). Previous studies reported that ring-porous species initiated wood growth earlier and exhibited faster early-season radial growth than that of diffuse-porous and coniferous species, as they need to produce new vessel conduits annually before the leaf flush can be supplied with enough water (Takahashi et al., 2013). However, cessation of wood formation (fully lignification) was almost synchronized between ring- and diffuse-porous species (Yin et al., 2024). At Harvard Forest, white pine (non-porous) had the longest wood formation duration, followed by red oak (ring-porous) and red maple (diffuse-porous) (Chen et al., 2022). Conversely, D’Orangeville et al. (2022) found that diffuse-porous species completed diameter growth 16–18 days faster than other types despite later onset at the same study region. These paradoxical results may arise from methodological differences (dendrometers vs. micro-sampling), when the former cannot precisely identify the initiation and cessation of the growth period. Thus, comparative studies of coexisting tree species with contrasting wood anatomies on wood formation processes may vary inter-specifically and respond to local environmental conditions, which need further clarification.

Trees growth responsiveness to environmental signals likely varies across wood types due to differences in xylem conduit structure, size, as well as in the scaling to the water transport and resource allocation (Friend et al., 2022). Coniferous tracheids are smaller in diameter and shorter than angiosperm vessels. A recent study identified strongly divergent climate sensitivities in xylem growth between gymnosperms and angiosperms following extreme drought events (Leifsson et al., 2023). Gymnosperms showed persistent radial growth reduction for 2–3 years after droughts compared to one-year reduction for angiosperms, indicating weakened drought sensitivity of xylem growth to water availability in gymnosperms. In contrast, angiosperms exhibited stronger drought sensitivity, consistent with findings that vapour pressure deficit rather than temperature shaped intra-annual growth in temperate broadleaves (Tumajer et al., 2022). However, ring-porous oaks may be less drought-sensitive than diffuse-porous angiosperms (Bader et al., 2022), as sap flux sensitivity to soil drying was approximately 2.2-2.3 times higher in diffuse-porous than in the ring-porous species (Meinzer et al., 2013). Overall, wood formation is governed by environmental conditions, phenological processes, and physiological mechanisms, with species-specific variation driven by wood anatomy.

The timing sequences of stem growth and leaf phenology reflects carbon allocation within and among organs, is influenced by environmental and internal controls, and yet remains species-specific (Abramoff and Finzi, 2015). Meta-analyses indicate that wood anatomy is a key trait explaining inter-specific variation in coordination of wood formation and canopy phenology, particularly leaf out timing (Panchen et al., 2014). In deciduous species, ring-porous trees initiate xylem formation before leaf unfolding (Marchand et al., 2020), whereas diffuse-porous species generally commence growth after leaf appearance (Takahashi et al., 2013). This stem-growth prioritization in ring-porous species restores hydraulic pathways after winter embolism, and guarantee rapid water transport for new leaf expansion and transpiration (Michelot et al., 2012). In contrast, evergreen conifers show no consistent relationship between stem and leaf phenology (Perrin et al., 2017; Qian et al., 2024a). Although research demonstrates that wood formation and canopy phenology (leaf development) are inconsistently linked due to distinct regulatory mechanisms, studies concurrently addressing both primary and secondary growth remain scarce (Silvestro et al., 2025).

In this study, we monitored xylogenesis and leaf phenology over a growing season in 10 tree species representing different wood anatomical types under warm-temperate climate condition. Our aims were: (1) to quantify intra-annual xylogenesis variation (i.e. the timing, duration and rate) among wood anatomical types and identify climatic drivers; (2) to compare the chronological order between the timing of xylogenesis initiation and leaf out and test whether this potential relationship varied correlated to wood anatomy. We hypothesized that: (H1) xylogenesis process and its climatic drivers are linked to wood anatomical types. In addition, we assumed that ring-porous and diffuse-porous species, owing to its larger conduits (vessels and/or tracheids) (Zimmermann et al., 2021), will show stronger growth dependence on water availability than conifers. (H2) the linkage between xylogenesis initiation and leaf out exhibits species-dependent variability, which are also dependent on wood anatomy. Specifically, ring-porous and semi-ring-porous species may develop new vessels before leaf out, whereas diffuse-porous species form their new vessels after or synchronously with leaf out.

## 2 Materials and methods

### 2.1 Study site and field sampling

**Figure 1:**
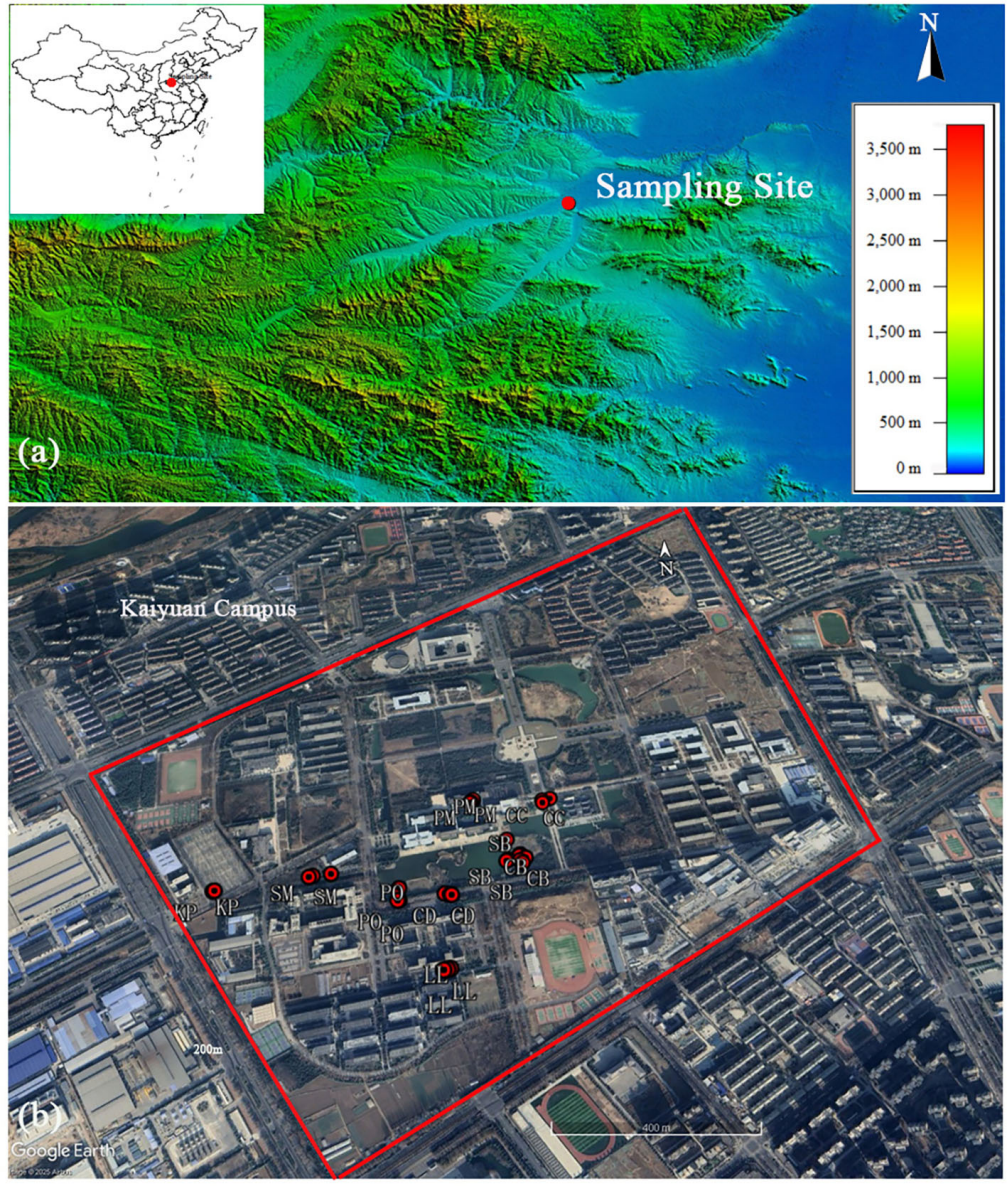
Geographical location of study site in central China **(a)** and the study area landscape from Google Earth **(b)**. Red points in b represent the sample trees. CD, *Cedrus deodara*; PM, *Pinus massoniana*; CB, *Catalpa bungei*; FC, *Fraxinus chinensis*; KP, *Koelreuteria paniculata*; CC, *Cinnamomum camphora*; LL, *Ligustrum lucidum*; PO, *Platanus occidentalis*; SB, *Salix babylonica*; SM, *Salix matsudana*.

The study was conducted on Kaiyuan Campus of Henan University of Science and Technology in Central China (34°36′N, 112°25′E, 142m a.s.l., **Figure 1**). The study site experiences a warm-temperate climate with the mean annual temperature of 14.2°C and the annual precipitation of 598 mm from 1960 to 2017. The study year of 2018 was relatively warm (+1.1°C) and wet (+223 mm) than the long-term average (1960-2017). In the study area, the herb layer is dominated by *Zoysia* spp. and shrubs layer are mainly by *Cercis chinensis* and *Pittosporum tobira*. The main soil type is Cinnamon soil (semi-Luvisols according to WRB system).

Ten tree species representing four wood anatomical types were selected, including two non-porous (conifers, *Cedrus deodara* and *Pinus massoniana*), three ring-porous (*Catalpa bungei*, *Fraxinus chinensis*, and *Koelreuteria paniculata*), two semi-ring-porous (*Cinnamomum camphora* and *Ligustrum lucidum*) and three diffuse-porous species (*Platanus occidentalis*, *Salix babylonica*, and *Salix matsudana*). All species are common species used for afforestation and greening in Central China (**Table 1**). Species information of wood anatomy, leaf trait and shade tolerance were obtained from ‘Flora of China’ (https://www.iplant.cn/foc), and wood anatomy was verified using ‘The Wood Database’ (https://www.wood-database.com/). Wood density values are derived from Global Wood Density Database (Chave et al., 2009; Zanne et al., 2010).

**Table 1:** Summary of 10 tree species.

Species	Wood porosity	Leaf trait	Shade tolerance	Wood density (g∙cm^-3^)	No. of trees	DBH (cm)	Height (m)	Age (years)
*Cedrus deodara*	Non-porous	Evergreen	Mid-Tolerant	0.44	3	19.8 (0.8)	12.1 (0.1)	16.0 (0.6)
Pinus massoniana	Non-porous	Evergreen	Intolerant	0.48	3	22.4 (0.5)	13.3 (0.4)	29.0 (3.2)
Catalpa bungei	Ring-porous	Deciduous	Intolerant	0.52	3	16.8 (0.5)	8.9 (0.2)	14.0 (1.0)
*Fraxinus chinensis*	Ring-porous	Deciduous	Mid-Tolerant	0.56	3	13.7 (0.3)	6.4 (0.1)	15.5 (0.5)
*Koelreuteria paniculata*	Ring-porous	Deciduous	Intolerant	0.62	2	22.8 (0.9)	9.3 (0.3)	19.0 (0.0)
*Cinnamomum camphora*	Semi-ring-porous	Evergreen	Intolerant	0.50	3	28.1 (1.0)	12.1 (0.4)	18.7 (2.6)
*Ligustrum lucidum*	Semi-ring-porous	Evergreen	Mid-Tolerant	0.63	3	13.6 (0.5)	5.9 (0.1)	20.0 (1.0)
*Platanus occidentalis*	Diffuse-porous	Deciduous	Intolerant	0.45	3	19.8 (1.3)	13.3 (0.9)	17.3 (1.3)
*Salix babylonica *	Diffuse-porous	Deciduous	Intolerant	0.36	3	35.3 (1.3)	11.1 (0.1)	18.3 (3.5)
*Salix matsudana*	Diffuse-porous	Deciduous	Intolerant	0.47	3	25.5 (1.4)	10.9 (0.3)	13.3 (0.9)

Wood density values are derived from Global Wood Density Database (Chave et al., 2009, Zanne et al., 2010). The values of diameter at breast height (DBH), tree height and age are mean ± standard error.

Three trees per species were selected, with similar ages, diameters, and heights. From March to December 2018, two or three microcores were collected from each tree at 7–10 day intervals around the stem at breast height (1.3 m) using a Trephor tool (Rossi et al., 2006a). The microcores were 2 mm in diameter and 15 mm in length, containing the previous one to three xylem rings, cambium zone and the adjacent phloem. In the laboratory, the microcores were dehydrated through ethanol series (50%, 70%, 90%, 95% and 100%), xylene, and embedded in paraffin. Transverse sections of 8 *μm* were cut using a Leica RM 2235 rotary microtome (Leica Microsystems, Wetzlar, Germany) and stained with 1% safranin and 0.5% fast green (in 95% ethanol) (Ding et al., 2021).

### 2.2 Microscopic observations and critical dates of xylem formation

Sections were examined under the Leica microscope (Leica DM 2500, Leica Microsystems, Wetzlar, Germany) to identify four xylogenesis stages, including the (i) onset of cell enlargement, (ii) onset of cell maturation, (iii) end of cell enlargement, and (iv) end of cell wall lignification. These four stages were identified following the methods of Rossi et al. (2006b) for conifers and Zhang et al. (2019b, 2022) for angiosperms. Each development stages dates were recorded as the day of the year (DOY) when at least 50% of the counted radial files showed the target phase for each species. The duration of cell production was identified as the period between the onset and end of cell enlargement. The duration of xylogenesis was defined as the period from the onset of cell enlargement to the end of cell wall lignification (He et al., 2016).

### 2.3 Intra-annual xylem formation fitting

For each section, the number of cell layers in the cambial zone was counted, and the new-formed xylem width was measured along three radial files using Image J. The width of new-formed xylem was standardized based on the previous ring width according Gruber et al. (2009). To assess the dynamics of xylem formation, the new-formed xylem width was modeled for each individual tree with a Gompertz function (Miller et al., 2022), which was defined as:


$$Y=Ae^{-e^{\left[-\kappa \left(x-x_{c}\right)\right]}}$$

Where *Y* is the intra-annual cumulative xylem width, *x* is the time expressed as DOY (day of the year), *A* is upper asymptote, *k* is the rate of change of the shape and *x_c_* is the date of the inflection point. In addition, the ring width of xylem growth was estimated by *A*, and the date of the maximum xylem growth rate (*r_max_*) was corresponded to the date of the inflection point (*x_c_*). The maximum xylem growth rate (*r_max_*) and the average growth rate (*r_mean_*) were computed as kA/e and (940)e·rmax, respectively (Rathgeber et al., 2011). To compare the xylem formation among different wood anatomical types, daily xylem growth increment and growth rate (fitting value) for each individual tree were standardized and converted in the form of percentage of final width.

### 2.4 Leaf phenology

Bud and leaf phenology was monitored for the same trees at 3–5 day intervals during spring and autumn. Three north-facing and three south-facing branches per tree were selected in the bottom part of the canopy. On each branch, the phases of bud development were recorded on terminal buds (Zhang et al., 2019a). Leaf unfolding (50% bud break) and leaf fall (50% leaf shed) dates were determined by linear interpolation and recorded as DOY (Vitasse et al., 2017).

### 2.5 Climate data

Daily climate data were obtained from the nearest state meteorological station in Mengjin, which was located approximately 20 km from the site (34°49′N, 112°26′E, 333m a.s.l.). These data were corrected and assessed with high quality of less than 1% missing rate and nearly 100% accuracy, which could be acquired from China Meteorological science data sharing network (http://data.cma.cn). The climate variables including mean air temperature (°C), mean ground surface temperature (°C), precipitation (mm), relative humidity (%), and sunshine duration hours (h) were selected. Daily vapor pressure deficit (VPD) was calculated from daily mean values of air temperature and relative humidity (Camarero, 2022). Photoperiod was used the data of sunshine duration hours.

### 2.6 Data analysis

For each stage of xylem formation, the date differences (onset, end, and duration) among four wood anatomical types were compared with the Kruskal–Wallis test due to a lack of normality and homoscedasticity of the data, and an ANOVA could not be conducted (Zhang et al., 2018). Subsequently, Mann–Whitney rank-sum test were performed for multiple comparisons test with SPSS (SPSS Inc., Chicago, IL, USA).

The effects of climate variables and wood anatomy on xylem growth increment and xylem growth rate were analyzed separately using linear mixed-effects models (LMMs) implemented in the *nlme* package in the R statistical software (Jevšenak et al., 2022; R Core Team, 2017). First, to reduce the collinearity problem, we calculated variance inflation factors (VIF) for all climate variables and excluded those with high collinearity according to the threshold of VIF values higher than 3.0. Ground surface temperature was excluded due to high collinearity (VIF > 10). Second, the xylem growth increment and xylem growth rate (Gompertz model fitting value), and precipitation data were log-transformed to fit the normal distribution.

In the model, the xylem growth increment and growth rate from each of the two sampling intervals were taken as dependent variables. Concurrent means (sums for precipitation) of climate variables and wood anatomy were included as fixed factors, with individual trees nested in tree species as a random factor (**Supplementary Table S4**). Furthermore, based on the initial results, anatomy-specific LMMs were subsequently run for each anatomy type. These models used all climate variables as fixed factors and individual trees as random factor (**Supplementary Table S5**, **Supplementary Table S6**). Finally, the selection of significant climate variables as fixed effects were based on Akaike’s Information Criterion (AIC), where model with the lowest AIC values was retained (**Supplementary Table S7**).

## 3 Results

### 3.1 Dynamics of xylem formation

**Figure 2:**
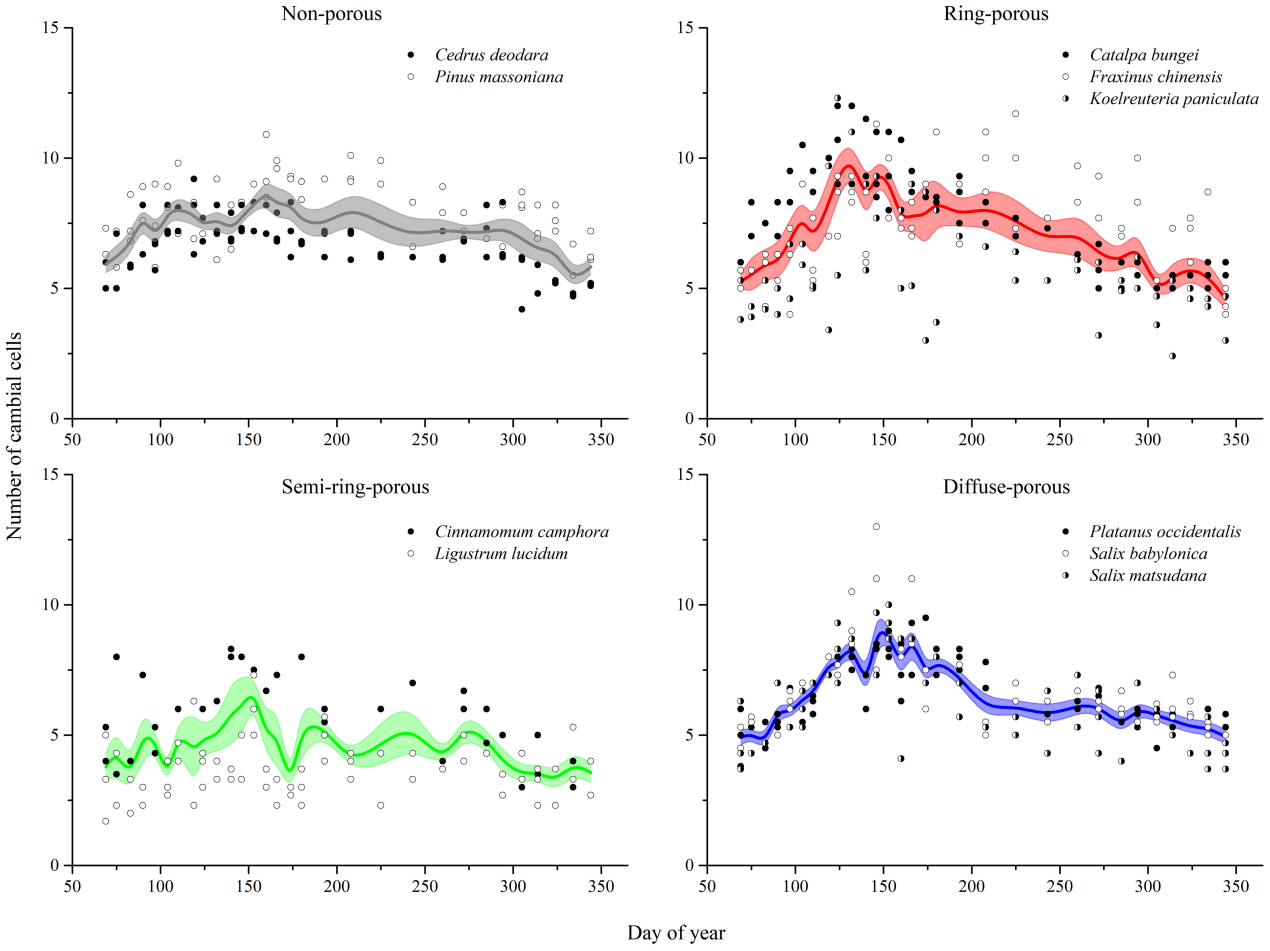
Number of cambial cells in 2018 across individuals (points) and averaged per non-porous (coniferous), ring-porous, semi ring-porous and diffuse-porous wood type (colored lines and bands). Lines represent mean values of cambial cells of all individual trees per wood type; horizontal bands depict the standard error ranges of individual trees in cambial cells.

During the study period, cambial activity in all species showed distinct seasonal patterns (**Figure 2**). Before the growing season, the dormant cambium contained 4–6 cell layers, with coniferous having the most cells (5.9 ± 0.3, mean ± SE) and semi-ring-porous trees the lowest (3.8 ± 0.4). In spring, cambial cells swelled and expanded radially. Ring-porous species achieved most cambial cells (10.1 ± 1.0) and reached peak cambial growth earliest on 12 May, preceding coniferous, semi-ring-porous, and diffuse-porous species by 28 days, 21 days and 14 days, respectively.

**Figure 3:**
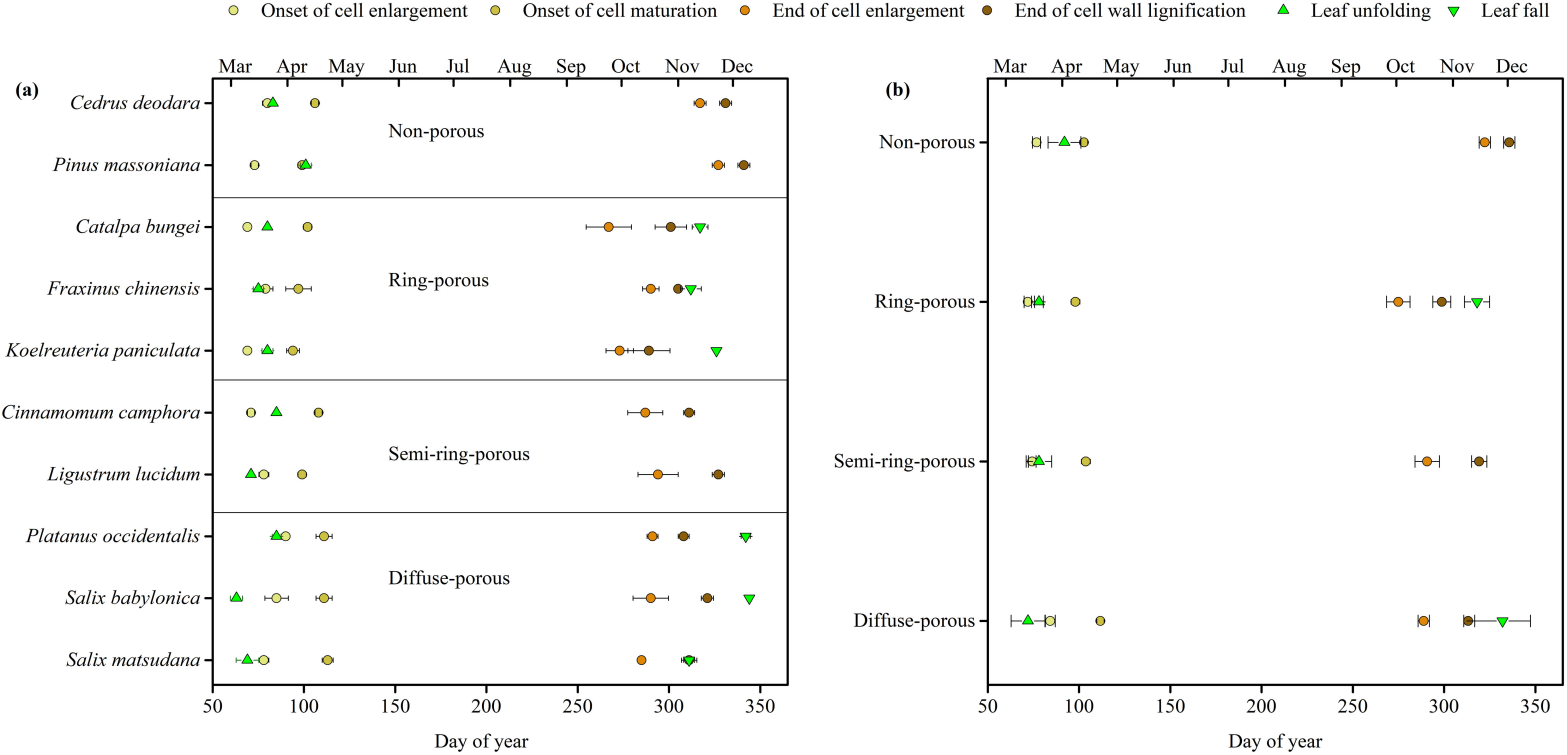
Dates of occurrence of xylem formation and leaf phenology for **(a)** 10 tree species and **(b)** four wood types (nonporous (coniferous), ring-porous, semi ring-porous and diffuse-porous). Error bars indicate the standard error between trees or types.

Intra-annual xylem formation differed markedly among anatomical types (**Figure 3**; **Supplementary Tables S1**, **S2**). On average, diffuse-porous trees started cambial activity (phase 1) on 25 March (DOY 84), notably later than coniferous, ring-porous and semi-ring-porous by 7 days, 12 days and 10 days, respectively. However, these differences diminish over the growing season due to higher growth rates in diffuse-porous trees compared to conifers. Cambial activity ended (phase 3) in the first half of October for ring-porous, semi-ring-porous, and diffuse-porous trees, which was approximately two weeks earlier than coniferous ones (18 November, DOY 322 ± 3.1, mean ± SE). The end of cell wall lignification (phase 4) occurred earliest in ring-porous trees (26 October, DOY 299 ± 4.9) and latest in conifers (2 December, DOY 336 ± 3.1).

**Figure 4:**
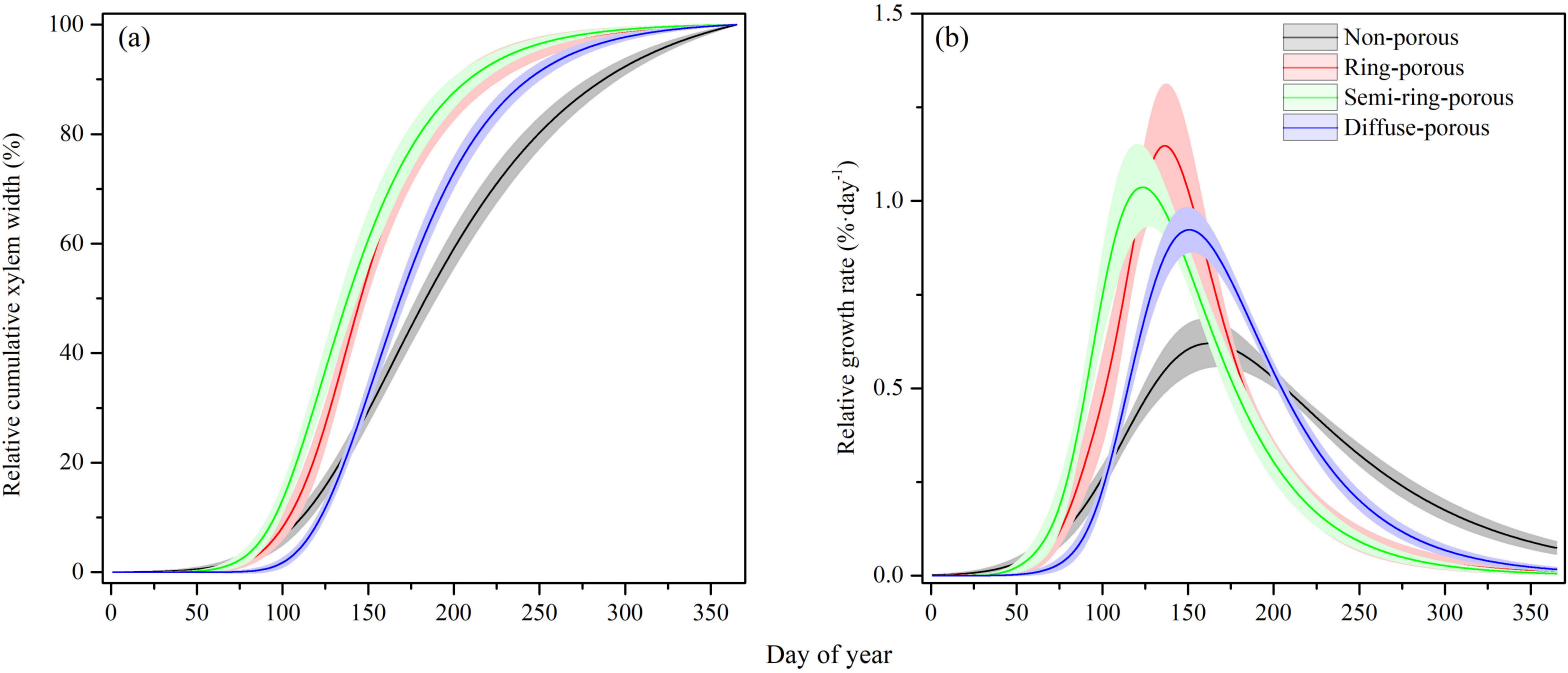
Xylem formation modeled by Gompertz function among four wood anatomical types, including nonporous (coniferous), ring-porous, semi-ring-porous and diffuse-porous. **(a)** Standardized xylem growth increment and **(b)** Standardized daily growth rate are averaged per each wood types. Horizontal bands represent the standard error ranges per wood type.

Consequently, broadleaved trees exhibited higher xylem radial growth rates and shorter duration of xylem formation than conifers (**Figure 4**; **Supplementary Figures S1**, **S2**). Ring-porous trees achieved the highest peak growth rates while conifers showed the lowest with maximum daily rates of 86 ± 11.2 *μm* and 34 ± 3.3 *μm* (mean ± SE), respectively (**Supplementary Table S3**). Peak xylem growth rate occurred earlier in ring-porous and semi-ring-porous trees (first half of May), followed by diffuse-porous trees (early June), and coniferous trees (mid-June).

### 3.2 Leaf phenology

Based on field observations, broadleaved species started leaf unfolding approximately two weeks earlier than conifers (**Figure 3**; **Supplementary Tables S1**, **S2**). Diffuse-porous species showed the earliest leaf unfolding on 13 March, especially for the *Salix* spp, followed by ring-porous and semi-ring-porous at the same time on 19 March. Compared to stem xylem growth, leaf unfolding in diffuse-porous species preceded xylem growth (corresponding to the onset of enlargement cell) by 15 days on average. In semi-ring-porous species, leaf unfolding coincided with xylem growth within 4 days, whereas in ring-porous and conifer species, leaf phenology lagged behind xylem growth by 6 days and 15 days, respectively.

### 3.3 Effects of climate variables on xylem growth

Linear mixed-effects models revealed divergent climate responses among wood anatomical types (**Supplementary Table S4**). Overall, air temperature predominantly influenced daily growth variables (increment and rate) in conifers, while precipitation played a dominant role in broadleaved species (**Table 2**; **Supplementary Tables S5**-**S7**).

**Table 2:** Parameter estimates for the selected linear mixed-effects models fitted to explain the variations of daily xylem growth increment and growth rate.

Wood anatomy		Xylem growth increment				Xylem growth rate				
		Estimate	SE	*t*-value	*p*-value		Estimate	SE	*t*-value	*p*-value
Non-porous	Intercept	0.190	0.048	3.928	<0.001	Intercept	-0.852	0.043	-19.612	<0.001
	TA	0.051	0.003	16.356	<0.001	TA	0.036	0.002	15.564	<0.001
	PHO	-0.043	0.007	-7.043	<0.001	PHO	-0.020	0.007	-3.094	0.002
Ring-porous	Intercept	0.293	0.154	1.904	0.071	Intercept	-0.947	0.212	-4.476	<0.001
	TA	0.077	0.007	11.681	0.000	PRE	0.657	0.090	7.330	<0.001
	PRE	0.202	0.076	2.649	0.009	PHO	0.186	0.021	8.797	<0.001
Semi- ring-porous	Intercept	0.088	0.310	0.283	0.811	Intercept	-0.904	0.320	-2.825	0.119
	TA	0.042	0.009	4.806	<0.001	TA	0.033	0.010	3.288	0.001
	PRE	0.330	0.089	3.706	<0.001	PRE	0.331	0.102	3.252	0.001
	PHO	0.085	0.024	3.513	0.001	PHO	0.117	0.028	4.221	<0.001
Diffuse-porous	Intercept	1.048	0.185	5.662	<0.001	Intercept	-0.367	0.091	-4.047	<0.001
	TA	0.088	0.006	15.782	<0.001	TA	0.076	0.004	18.675	<0.001
	PRE	0.223	0.070	3.189	0.002	PRE	0.124	0.048	2.601	0.01
	PHO	-0.050	0.048	3.928	0.002					

Linear mixed-effects models were performed separately for xylem growth and rate for each wood anatomy, with climate variables as fixed factors and individual trees as random factor. TA, air temperature; PRE, precipitation; RH, relative humidity; VPD, vapor pressure deficit; PHO, photoperiod.

Air temperature positively affected xylem growth increment across all wood types, but significantly influenced growth rates only in conifers and diffuse-porous species. This indicates a stronger response of growth increment to air temperature than that of growth rate. Precipitation had no significant effect on conifer growth (increment: *p* = 0.530, **Supplementary Table S5**; rate: *p* = 0.188, **Supplementary Table S6**). In contrast, both radial growth increment and growth rate in broadleaved species were primarily driven by precipitation (*p<* 0.05 for all three wood types), with stronger correlations in ring-porous and semi-ring-porous species than in diffuse-porous species. Moreover, photoperiod (PHO) effect on growth varied considerably across tree species. Specifically, the PHO correlated negatively with growth variables in conifers and diffuse-porous species but positively for ring-porous and semi-ring-porous species.

## 4 Discussion

### 4.1 Earlier onset and faster growth rate in (semi-)ring-porous species

Consistent with our first hypothesis (H1), there were substantial differences in xylem formation among trees having different wood anatomical types (**Figures 3**, **4**). Ring-porous species exhibited earlier growth onset and shorter growth duration, coupled with faster xylem growth rates. Diffuse-porous species initiated growth later but achieved higher growth rates than conifers, enabling earlier completion of xylogenesis in November. In contrast, evergreen conifers showed the longest growth duration and lowest growth rates.

These patterns align with fundamental differences in wood anatomy and functionality between conifers and broadleaved species (Islam et al., 2018; Sperry et al., 1994). Consistently, previous studies have documented that ring-porous species typically display intensive xylem formation, while diffuse-porous species and conifers show less intensive xylem production (Qian et al., 2024b; Takahashi et al., 2015), which are likely reflecting divergent resource allocation strategies across the acquisitive-conservative spectrum. Ring-porous species with more rapid water transport and higher leaf photosynthetic rate traits, occurring at the fast-acquisitive side of the spectrum and the diffuse-porous or coniferous species located on the opposite side (Pompa-García et al., 2023; Yin et al., 2023). For example, Foster (2017) synthesized 37 defoliation studies and reported 40–100% growth reductions in diffuse-porous species and evergreen conifers (versus 10-40% in ring-porous *Quercus* and deciduous conifers), attributed to lower non-structural carbohydrate (NSC) storage making them more vulnerable to mortality when stress accumulates, which align well with our findings.

Critically, in our study, the delayed onset of xylem growth (*ca.* 12 days) in diffuse-porous species is partially offset by theirs higher growth rate after May, resulting in comparable growth duration and growth rate between ring-porous and diffuse-porous species (**Figure 3**; **Supplementary Tables S2**, **S3**). However, some studies suggest that wood formation in diffuse-porous species generally begins later and lasts shorter than in ring-porous species (Chen et al., 2022; Qian et al., 2024a; Werf et al., 2007). This partial discrepancy in growth duration may be relate to the differences in local climatic conditions and species-specific growth-climate responses. Unlike the warm temperate climate of our study site, these studies were mainly conducted in temperate climate regions. In colder region, the length of the growing season required for completely xylem differentiation is likely shortened due to temperature limitation. Additionally, diffused-porous species may exhibit greater inter-annual variability in radial growth compared to ring-porous species (Chen et al., 2022). For instance, red maple (*Acer rubrum*) showed second peak growth due to the high water supply during the late growing season, leading to the longer growth duration and the wider annual rings. This species-specific plastic response of diffuse-porous is potentially related to their high sensitivity of wood formation to water supply (D’Orangeville et al., 2022).

### 4.2 Divergent responses of radial growth to climatic factors

Environmental drivers of xylem formation are relatively well understood (Delpierre et al., 2019; Deslauriers and Morin, 2005; Güney et al., 2020). One of the remarkable findings in our study was that air temperature significantly influenced ring width across all species, whereas precipitation drove radial growth exclusively in broadleaved trees, with no significant effect in conifers (**Table 2**; **Supplementary Table S4**). Notably, precipitation sensitivity was markedly stronger in ring-porous and semi-ring-porous species than in diffuse-porous types. These results confirm H1, demonstrating that differences in xylem formation in response to climatic factors are specific to wood anatomy types.

Divergent drought sensitivities between gymnosperms and angiosperms are increasingly recognized (Leifsson et al., 2023). Our observed differences among wood anatomy species’ respond to precipitation might mirror differences in the radial growth and water relations (Zweifel et al., 2006). Ring-porous trees have large xylem conduits and its earlywood vessels account for more than 90% of water conductivity (Beeckman, 2016). However, these large vessels remain functional for only a single growing season, which enables trees to acquire enough water for early growth (García-González et al., 2016). This special need for water at the beginning of the growing season may make ring-porous species more sensitive to water supply in spring (Zhang et al., 2019b). For diffuse-porous species, they depend on relatively smaller and similar-sized vessels with greater embolism resistance, yet still exhibit significant water dependence due to their high photosynthetic demands (Serrano-Notivolia et al., 2025). Conifers with narrow tracheids providing high hydraulic safety margins that enhance drought resistance at the cost of reduced precipitation sensitivity (McCulloh et al., 2010), implying conifer species to be more resistant to drought-induced cavitation than broadleaved species. These functional differences align with whole-tree water use patterns predicted from sap flux models (Berdanier et al., 2016), reinforcing the primacy of xylem anatomy in governing hydraulic strategies (Camarero et al., 2025), corroborating our results.

Photoperiod, or day length, is another critical factor that regulates cambial activity, maximum growth rates, and growth cessation (Huang et al., 2018; Rossi et al., 2006c; Saderi et al., 2019). In this study region, photoperiod with positive correlations in (semi-)ring-porous species contrasting sharply with negative associations in conifers and diffuse-porous trees (**Table 2**; **Supplementary Tables S5**, **S6**). These divergences likely arise from species-specific growth patterns (Qian et al., 2024b) and interactions with other factors (Laube et al., 2014), especially photoperiod in combination with temperature (Huang et al., 2020), which alter photoperiod requirements throughout the growing season.

In spring, longer photoperiod and/or warmer temperatures may trigger an earlier cambial activity, while shorter photoperiod in autumn and cooler temperatures induce growth cessation (Way and Montgomery, 2014). Accordingly, (semi-)ring-porous species characterized by fast growth rates (earlier onset and cessation of growth; **Figure 3**) are believed to respond positively to photoperiod in spring and summer, as longer photoperiod and/or warmer temperatures may advance growth during the acceleration phase. Contrarily, conifers with slower growth rate (later cessation) may not benefit from extended growing season under warmer climate, especially as the autumn phenological events (growth cessation and bud set) (Morin et al., 2010). Mu et al. (2023) showed a negative effect of photoperiod on coniferous wood formation cessation. Their study indicated that longer photoperiod was associated with earlier cessation and potentially reduced radial growth, compiling a dataset from 62 sites across mid- and high-latitudes in the Northern Hemisphere. Although photosynthesis is enhanced under longer day lengths, resource allocation may favor other growth processes, such as height or needle growth rather than radial growth (Basler and Körner, 2014), which is proposed to explain this negative relationship between photoperiod and radial growth. Other observations confirmed that longer photoperiod and warmer temperature extended the growing season of spruce in both bud and stem, but with reduced starch storage reserves relative to that attained under shorter photoperiod (Hamilton et al., 2016).

### 4.3 Temporal sequences between xylem formation and leaf out phenology in relation to wood anatomy

The sequence of xylem and leaf phenology helps clarify physiological mechanisms governing tree growth process (Arend et al., 2024; Zhang et al., 2019a). Our results demonstrate that diffuse-porous species produced new xylem cells after leaf development, whereas ring-porous, semi-ring-porous, and coniferous species initiated xylem formation prior to leaf unfolding (**Figure 3**; **Supplementary Tables S1**, **S2**). This expected pattern aligns with long-established linkages between radial growth and leaf phenology (Gričar et al., 2022; Rossi et al., 2009).

A proposed explanation for the similar developmental sequences in (semi-)ring-porous and coniferous species, which differs from that in diffuse-porous species, is assumed to involve carbon allocation dynamics during wood formation. Ring-porous species appear to rely substantially on stored carbon from previous years for earlywood growth (Zweifel et al., 2006). Consistently, conifers were capable to use the substrates from older foliage to initiate xylem growth which is agreement with their later bud break (Michelot et al., 2012). In contrast, diffuse-porous species like beech may supply resources for wood construction entirely through current-year assimilates (Barbaroux et al., 2003). Supporting this, Palacio et al. (2011) used *δ*^13^C and quantified the relative contribution of stored carbon to wood formation, which was greater in the ring-porous (55–70%) than in the diffuse-porous species (35–60%), although each species followed different seasonal patterns.

Another explanation might be that leaf out time correlates with xylem anatomy that can impact xylem water transport capacity in spring. Plants with narrow conduits that experience limited embolism will maintain high xylem conductance, enabling early new canopy support in spring. In contrast, plants with wide conduits experiencing reduced xylem conductance must wait for new conduits formation before leaf out (Savage et al., 2022). Our climate-growth analysis nonetheless confirms that water availability (precipitation) exerts stronger influence on (semi-)ring-porous trees than diffuse-porous and coniferous trees (**Table 2**). Ring-porous trees with large earlywood vessels, whose water relations heavily rely on current-year xylem; therefore, they need to produce a new set of xylem vessels before bud burst to meet water transport demands so that newly emerging leaves can be supplied with water (Hacke and Sperry, 2001). In contrast, diffuse-porous vessels, with smaller embolism-resistant vessels (Pittermann and Sperry, 2005), primarily utilize previous-year vessels and thus leaf out earlier.

A third explanation involves differential auxin sensitivity. Auxin, especially indole-3-acetic acid, is considered as an important promoter of cambium reactivation which is produced by growing young leaves (Du and Yamamoto, 2007). Some studies revealed that diffuse-porous species require higher auxin thresholds for xylogenesis initiation than ring-porous species, causing them to produce vessels only until new leaves become active auxin sources (Aloni, 2015). Conversely, debudding experiments showed that bud development is non-essential for cambial reactivation in ring-porous hardwood (Kudo et al., 2014), while conifers might likely utilize the reserved auxin from dormant stem tissues for triggering cambial reactivation (Guo et al., 2022).

## 5 Conclusion

As hypothesized, we found that the seasonal dynamics of xylem radial growth differed across wood anatomy, which both (semi-)ring-porous and diffuse-porous trees display shorter growing periods and faster growth rates than coniferous trees (**Figures 3**, **4**; **Supplementary Table S2**). Radial growth responses to climatic factors varied across species with differing wood anatomies, where xylem growth of conifers correlated positively with air temperature but broadleaved trees were driven mainly by increasing precipitation (**Table 2**), reflecting species-specific differences in drought sensitivity (Grossiord et al., 2020; Lévesque et al., 2013). Corroborating our results, previous studies have found that ring-porous oaks are able to exploit soil water resources more effectively across broader soil moisture and atmospheric dryness ranges than either diffuse-porous angiosperms or conifers (Meinzer et al., 2013). These divergent water-use responses among studied species are consistent with their distributions along geographical gradients of water availability.

Moreover, we demonstrated inconsistent connections between xylogenesis initiation and leaf expansion (unfolding) across wood anatomical types. This finding explains why seasonal dynamics of woody biomass cannot be quantified through eddy covariance or satellite observations alone, given that leaf phenology is independent from xylem phenology (Chen et al., 2022; Wang et al., 2025a). Therefore, studying temporal relationships between wood formation and leaf phenology (also known as canopy phenology) remains essential, not only for conifers but also for broadleaves, to meet future modelling requirements. Crucially, wood formation rather than leaf development constitutes the primary biological process through which carbon is durably sequestered in woody plants. Thus, although single-year observation may limit the generalizability of findings, wood anatomy emerges as a key functional trait providing fundamental insights for interpreting variability in tree growth and carbon sequestration (Simovic et al., 2024).

## Data Availability

The original contributions presented in the study are included in the article/**Supplementary Material**. Further inquiries can be directed to the corresponding author.
