# Supplementary material for: Contrasting wood anatomy drives divergent xylogenesis and climate responses in 10 warm-temperate trees
Source: Front Plant Sci. 2026 Jan 16;16:1660428. doi: 10.3389/fpls.2025.1660428 (PMC12856763; doi:10.3389/fpls.2025.1660428)
Supplement: Supplementary file 1 [file DataSheet1.docx]

**Supplement materials**


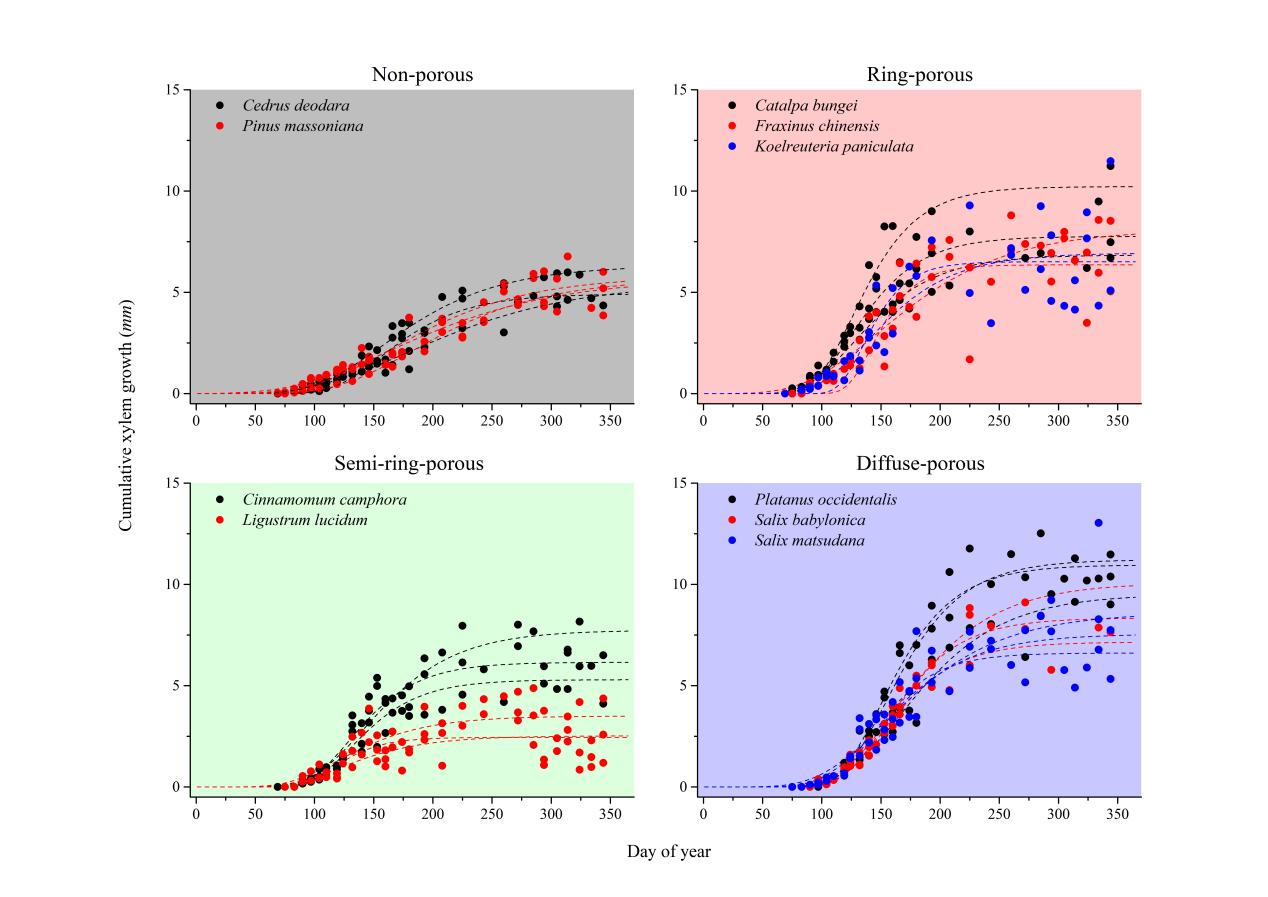
Figure S1 Intra-annual xylem growth dynamics of four wood anatomical types in 2018. Dots and dash lines represent the measured values and fitted values by the Gompertz function across individuals, respectively.


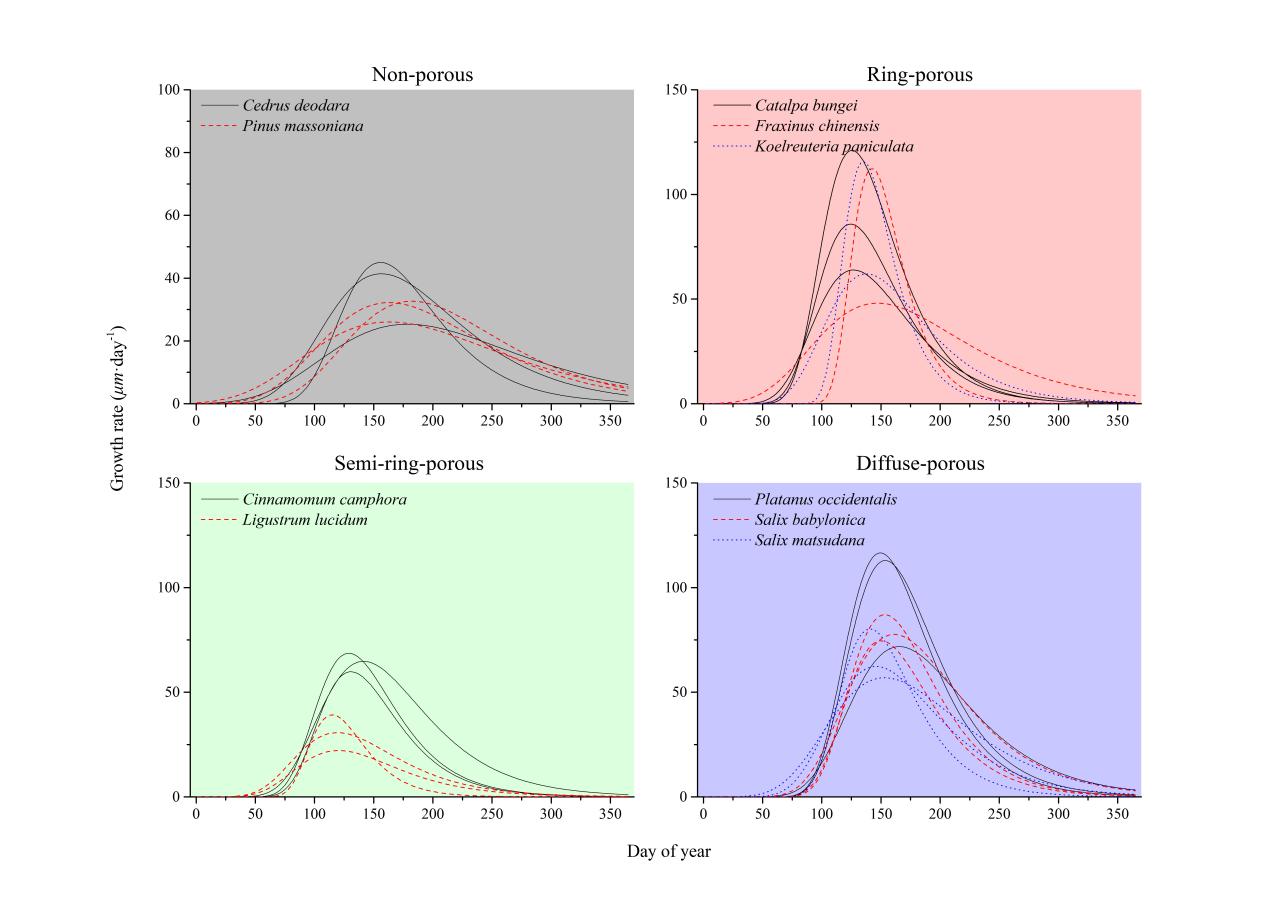


Figure S2 Intra-annual xylem daily growth rate of four wood anatomical types in 2018. Lines represent the fitted values by the Gompertz function across individuals.

| Table S1. Phases of xylem formation and leaf phenology for 10 tree species. | | | | | | | | | |
| --- | --- | --- | --- | --- | --- | --- | --- | --- | --- |
| Wood anatomy | Species | Onset of cell enlargement  (phase 1, DOY) | Onset of cell maturation  (phase 2, DOY) | End of cell enlargement (phase 1, DOY) | End of cell maturation  (phase 1, DOY) | Duration of cell production  (phase 1-3, days) | Duration of xylogenesis  (phase 1-4, days) | Leaf unfolding  (DOY) | Leaf fall  (DOY) |
| Non-porous | *Cedrus deodara* | 80 (2.7) | 106 (2.0) | 317 (3.3) | 331 (3.3) | 237 (3.1) | 250 (0.7) | 83 (0.0) |  |
|  | *Pinus massoniana* | 73 (2.0) | 99 (2.3) | 327 (3.3) | 341 (3.3) | 254 (3.0) | 268 (4.7) | 101 (3.0) |  |
| Ring-porous | *Catalpa bungei* | 69 (0.0) | 102 (2.3) | 267 (12.4) | 301 (8.6) | 198 (12.4) | 232 (8.6) | 80 (0.0) | 317 (4.3) |
|  | *Fraxinus chinensis* | 79 (4.0) | 97 (7.0) | 290 (4.5) | 305 (0.0) | 211 (0.5) | 226 (4.0) | 75 (2.8) | 312 (5.7) |
|  | *Koelreuteria paniculata* | 69 (0.0) | 94 (3.5) | 273 (7.5) | 289 (11.5) | 204 (7.5) | 220 (11.5) | 80 (1.0) | 326 (0.0) |
| Semi-ring-porous | *Cinnamomum camphora* | 71 (2.0) | 108 (2.0) | 287 (9.6) | 311 (3.0) | 216 (10.0) | 240 (5.0) | 85 (0.0) |  |
|  | *Ligustrum lucidum* | 78 (2.7) | 99 (2.3) | 294 (11.0) | 327 (3.3) | 216 (9.9) | 250 (5.2) | 71 (0.0) |  |
| Diffuse-porous | *Platanus occidentalis* | 90 (0.0) | 111 (4.4) | 291 (3.0) | 308 (3.0) | 201 (3.0) | 218 (3.0) | 85 (2.4) | 342 (2.5) |
|  | *Salix babylonica* | 85 (6.4) | 111 (4.4) | 290 (9.7) | 321 (3.3) | 205 (8.8) | 236 (6.8) | 63 (3.3) | 344 (0.0) |
|  | *Salix matsudana* | 78 (2.7) | 113 (3.0) | 285 (0.0) | 311(3.0) | 207 (2.7) | 233 (2.8) | 69 (6.2) | 311 (4.2) |
| Phases values represent mean (standard error) for the given day of year (DOY); duration values represent the days. | | | | | | | | | |

| Table S2. Phases of xylem formation and leaf phenology averaged per non-porous (coniferous), ring-porous, semi-ring-porous or diffuse-porous wood anatomy. | | | | | | | | | | |
| --- | --- | --- | --- | --- | --- | --- | --- | --- | --- | --- |
| Wood anatomy | No. of species | No. of trees | Onset of cell enlargement (phase 1, DOY) | Onset of cell maturation  (phase 2, DOY) | End of cell enlargement  (phase 3, DOY) | End of cell wall lignification  (phase 4, DOY) | Duration of cell production  (phase 1-3, days) | Duration of xylogenesis  (phase 1-4, days) | Leaf unfolding (DOY) | Leaf fall (DOY) |
| Non-porous | 2 | 6 | 77 (2.2)^ab^ | 103 (2.0)^b^ | 322 (3.1)^a^ | 336 (3.1)^a^ | 246 (4.3)^a^ | 259 (4.4)^a^ | 92 (9.0)^a^ |  |
| Ring-porous | 3 | 8 | 72 (2.0)^b^ | 98 (2.4)^b^ | 275 (6.4)^b^ | 299 (4.9)^c^ | 203 (5.4)^b^ | 227 (4.7)^c^ | 78 (2.4)^b^ | 318 (6.9)^b^ |
| Semi-ring-porous | 2 | 6 | 74 (2.1)^b^ | 104 (2.4)^b^ | 291 (6.7)^b^ | 319 (4.2)^b^ | 216 (6.3)^b^ | 245 (3.9)^b^ | 78 (7.0)^b^ |  |
| Diffuse-porous | 3 | 9 | 84 (2.7)^a^ | 112 (2.0)^a^ | 289 (3.1)^b^ | 313 (2.5)^b^ | 205 (2.9)^b^ | 229 (3.6)^c^ | 72 (9.3)^b^ | 332 (15.3)^a^ |
| Phases values represent mean (standard error) for the given day of year (DOY); duration values represent the days; Letters (a,b) indicate significant differences between four wood anatomical types based on Mann-Whitney rank-sum Test (*p*<0.05) | | | | | | | | | | |

| Table S3. Parameters (*A*, *t*p, *k*) of the Gompertz functions fitted to the stem intra-annual xylem growth per species and averaged per wood anatomy | | | | | | | |
| --- | --- | --- | --- | --- | --- | --- | --- |
| Species | Measure value (*μm*) | *A* (*μm*) | *t*_p_ (DOY) | *k* | *r_max_* (*μm* ∙ day^-1^) | *r_m_* (*μm* ∙ day^-1^) | Adj. *r*^2^ |
| *Cedrus deodara* | 5416.7 (336.6) | 5591.0 (406.5) | 164 (7.5) | 0.0184 (0.0036) | 37.23 (6.05) | 22.77 (3.70) | 0.915-0.966 |
| *Pinus massoniana* | 6230.7 (318.9) | 5743.0 (41.6) | 169 (6.4) | 0.0141 (0.0010) | 29.88 (2.14) | 18.28 (1.31) | 0.888-0.917 |
| *Catalpa bungei* | 8987.1 (1123.5) | 8274.9 (1007.6) | 126 (0.7) | 0.0292 (0.0020) | 90.24 (16.64) | 55.19 (10.18) | 0.933-0.947 |
| *Fraxinus chinensis* | 8660.3 (137.6) | 7234.2 (876.9) | 145 (2.3) | 0.0321 (0.0160) | 80.26 (32.21) | 49.09 (19.70) | 0.772-0.802 |
| *Koelreuteria paniculata* | 10381.8 (1093.1) | 6726.1 (213.8) | 136 (1.3) | 0.0363 (0.0120) | 88.84 (26.83) | 54.34 (16.41) | 0.692-0.770 |
| *Cinnamomum camphora* | 7689.4 (376.4) | 6398.1 (716.6) | 134 (3.9) | 0.0279 (0.0026) | 64.39 (2.56) | 39.38 (1.56) | 0.845-0.907 |
| *Ligustrum lucidum* | 4679.1 (115.6) | 2825.7 (339.3) | 118 (1.6) | 0.0304 (0.0066) | 29.96 (5.01) | 18.32 (3.06) | 0.409-0.531 |
| *Platanus occidentalis* | 11105.4 (717.9) | 10567.4 (526.2) | 156 (4.8) | 0.0256 (0.0026) | 100.64 (13.62) | 61.56 (8.33) | 0.942-0.960 |
| *Salix babylonica* | 8488.1 (356.8) | 8523.9 (849.0) | 155 (3.2) | 0.0259 (0.0025) | 79.78 (3.75) | 48.80 (2.29) | 0.943-0.971 |
| *Salix matsudana* | 9681.0 (1684.7) | 7592.5 (576.8) | 147 (3.6) | 0.0245 (0.0044) | 66.49 (7.03) | 40.67 (4.30) | 0.743-0.908 |
| Non-porous | 5823.7 (275.9) | 5667.0 (185.9) | 166 (4.6) |  | 33.56 (3.3) | 20.52 (2.0) |  |
| Ring-porous | 9343.1 (556.0) | 7411.7 (508.3) | 136 (3.4) |  | 86.45 (11.2) | 52.87 (6.9) |  |
| Semi-ring-porous | 6184.2 (695.8) | 4611.9 (874.0) | 126 (4.0) |  | 47.17 (8.1) | 28.85 (5.0) |  |
| Diffuse-porous | 9758.2 (658.1) | 8894.6 (551.2) | 153 (2.5) |  | 82.31 (6.7) | 50.34 (4.1) |  |
| *A* = the upper asymptote of the total xylem growth, *t_p_* = the date of maximum growth rate occurrence (the inflection point), *r_max_* = the maximum growth rate, *r_m_ =* the average growth rate, and Adj.*R*^2^ = Adjusted *R*^2^, ******* correspond to a *p* < 0.001; Values represent mean (standard error). | | | | | | | |

| Table S4. Summary of linear mixed-effects models on daily xylem growth increment and growth rate. Climate variables and wood anatomy were designated as fixed factors, whereas individual trees nested in tree species as random factor. TA, air temperature; PRE, precipitation; RH, relative humidity; VPD, vapor pressure deficit; PHO, photoperiod. | | | | | | |
| --- | --- | --- | --- | --- | --- | --- |
| Model parameters | | Xylem growth increment | |  | Xylem growth rate | |
|  |  | *F* | *p*-value |  | *F* | *p*-value |
| Air temperature | Intercept | 34.23 | <0.001 |  | 52.41 | <0.001 |
|  | Wood anatomy | 3.24 | **0.049** |  | 3.73 | **0.03** |
|  | TA | 740.21 | **<0.001** |  | 591.45 | **<0.001** |
|  | Wood anatomy × TA | 22.62 | **<0.001** |  | 17.2 | **<0.001** |
| Precipitation | Intercept | 665.04 | <0.001 |  | 82.3 | <0.001 |
|  | Wood anatomy | 30.85 | **<0.001** |  | 30.1 | **<0.001** |
|  | PRE | 105.63 | **<0.001** |  | 59.21 | **<0.001** |
|  | Wood anatomy × PRE | 2.24 | 0.082 |  | 1.86 | 0.136 |
| Relative humidity | Intercept | 109.41 | <0.001 |  | 18.69 | **<0.001** |
|  | Wood anatomy | 7.96 | **<0.001** |  | 7.25 | **<0.001** |
|  | RH | 14.45 | **<0.001** |  | 2.04 | 0.153 |
|  | Wood anatomy × RH | 0.53 | 0.661 |  | 0.48 | 0.699 |
| Vapor pressure deficit | Intercept | 263.07 | <0.001 |  | 1.15 | 0.299 |
|  | Wood anatomy | 10.24 | **0.001** |  | 10.92 | **<0.001** |
|  | VPD | 133.31 | **<0.001** |  | 176.61 | **<0.001** |
|  | Wood anatomy × VPD | 6.21 | **<0.001** |  | 5.46 | **0.001** |
| Photoperiod | Intercept | 185.63 | <0.001 |  | 0.02 | 0.886 |
|  | Wood anatomy | 7.45 | **0.001** |  | 7.90 | **0.001** |
|  | PHO | 92.93 | **<0.001** |  | 136.39 | **<0.001** |
|  | Wood anatomy × PHO | 6.835 | **<0.001** |  | 6.45 | **<0.001** |

| Table S5. Summary of linear mixed-effects models on daily xylem growth increment. Linear mixed-effects models were performed for each wood anatomy, with climate variables as fixed factors and individual trees as random factor. TA, air temperature; PRE, precipitation; RH, relative humidity; VPD, vapor pressure deficit; PHO, photoperiod. | | | | | | |
| --- | --- | --- | --- | --- | --- | --- |
| Wood anatomy | Variable | Estimate | SE | df | *t*-value | *p*-value |
| Non-porous | Intercept | 0.202 | 0.210 | 170.772 | 0.961 | 0.338 |
|  | TA | 0.043 | 0.006 | 169.650 | 7.284 | **<0.001** |
|  | PRE | 0.027 | 0.043 | 169.650 | 0.629 | 0.530 |
|  | RH | -0.067 | 0.332 | 169.650 | -0.203 | 0.840 |
|  | VPD | -0.052 | 0.111 | 169.650 | -0.465 | 0.642 |
|  | PHO | -0.045 | 0.010 | 169.650 | -4.554 | **<0.001** |
| Ring-porous | Intercept | -0.444 | 0.600 | 194.331 | -0.739 | 0.461 |
|  | TA | 0.050 | 0.017 | 192.714 | 2.978 | **0.003** |
|  | PRE | 0.267 | 0.122 | 192.665 | 2.187 | **0.030** |
|  | RH | 0.923 | 0.947 | 192.685 | 0.974 | 0.331 |
|  | VPD | 0.363 | 0.314 | 192.653 | 1.157 | 0.249 |
|  | PHO | 0.039 | 0.028 | 192.669 | 1.409 | 0.161 |
| Semi-ring-porous | Intercept | 0.375 | 0.605 | 20.521 | 0.619 | 0.543 |
|  | TA | 0.043 | 0.015 | 169.188 | 2.817 | **0.005** |
|  | PRE | 0.403 | 0.111 | 169.188 | 3.619 | **<0.001** |
|  | RH | -0.532 | 0.860 | 169.188 | -0.619 | 0.536 |
|  | VPD | -0.011 | 0.287 | 169.188 | -0.039 | 0.969 |
|  | PHO | 0.076 | 0.025 | 169.188 | 2.999 | **0.003** |
| Diffuse-porous | Intercept | 1.185 | 0.350 | 258.943 | 3.382 | 0.001 |
|  | TA | 0.092 | 0.010 | 256.201 | 9.390 | **<0.001** |
|  | PRE | 0.230 | 0.071 | 256.201 | 3.214 | **0.001** |
|  | RH | -1.005 | 0.551 | 256.201 | -1.824 | 0.069 |
|  | VPD | -0.085 | 0.184 | 256.201 | -0.461 | 0.645 |
|  | PHO | -0.051 | 0.016 | 256.201 | -3.103 | **0.002** |

| Table S6. Summary of linear mixed-effects models on daily growth rate. Linear mixed-effects models were performed for each wood anatomy, with climate variables as fixed factors and individual trees as random factor. | | | | | | |
| --- | --- | --- | --- | --- | --- | --- |
| Wood anatomy | Variable | Estimate | SE | df | *t*-value | *p*-value |
| Non-porous | Intercept | -0.884 | 0.186 | 170.872 | -4.750 | <0.001 |
|  | TA | 0.031 | 0.005 | 169.413 | 6.031 | **<0.001** |
|  | PRE | 0.050 | 0.038 | 169.413 | 1.321 | 0.188 |
|  | RH | -0.022 | 0.294 | 169.413 | -0.075 | 0.940 |
|  | VPD | 0.095 | 0.098 | 169.413 | 0.964 | 0.336 |
|  | PHO | -0.021 | 0.009 | 169.413 | -2.446 | **0.015** |
| Ring-porous | Intercept | -1.677 | 0.655 | 195.252 | -2.559 | 0.011 |
|  | TA | 0.031 | 0.019 | 192.566 | 1.688 | 0.093 |
|  | PRE | 0.295 | 0.134 | 192.504 | 2.204 | **0.029** |
|  | RH | 1.255 | 1.037 | 192.530 | 1.210 | 0.228 |
|  | VPD | 0.591 | 0.344 | 192.489 | 1.720 | 0.087 |
|  | PHO | 0.069 | 0.030 | 192.510 | 2.276 | **0.024** |
| Semi-ring-porous | Intercept | -0.876 | 0.674 | 30.992 | -1.301 | 0.203 |
|  | TA | 0.024 | 0.017 | 169.262 | 1.385 | 0.168 |
|  | PRE | 0.419 | 0.127 | 169.262 | 3.296 | **0.001** |
|  | RH | -0.152 | 0.981 | 169.262 | -0.155 | 0.877 |
|  | VPD | 0.221 | 0.327 | 169.262 | 0.677 | 0.499 |
|  | PHO | 0.105 | 0.029 | 169.262 | 3.618 | **<0.001** |
| Diffuse-porous | Intercept | -0.110 | 0.369 | 259.246 | -0.297 | 0.767 |
|  | TA | 0.071 | 0.010 | 256.223 | 6.862 | **<0.001** |
|  | PRE | 0.245 | 0.075 | 256.223 | 3.250 | **0.001** |
|  | RH | -0.534 | 0.581 | 256.223 | -0.919 | 0.359 |
|  | VPD | 0.171 | 0.194 | 256.223 | 0.883 | 0.378 |
|  | PHO | -0.020 | 0.017 | 256.223 | -1.149 | 0.251 |

| Table S7. Model selections of linear mixed-effects models fitted on daily xylem growth increment and growth rate. Mixed-effects models were performed separately for growth increment and rate, with climate variables as fixed factors and individual trees as random factor. | | | | | |
| --- | --- | --- | --- | --- | --- |
| Wood anatomy | Dependent: Xylem growth increment | |  | Dependent: Xylem growth rate | |
|  | Model fixed effects | AIC |  | Model fixed effects | AIC |
| Non-porous | TA+PHO | -24.81 |  | TA+PHO | -64.30 |
|  | TA+PHO+PRE | -20.81 |  | TA+PHO+PRE | -59.56 |
| Ring-porous | TA+PRE | 422.94 |  | PRE+PHO | 470.12 |
|  | TA+PRE+PHO | 426.27 |  | PRE+PHO+VPD | 483.23 |
| Semi-ring-porous | TA+PRE+PHO | 321.21 |  | PRE+PHO | 367.45 |
|  | TA+PRE+PHO+RH | 319.00 |  | PRE+PHO+TA | 370.61 |
| Diffuse-porous | TA+PRE+PHO | 338.59 |  | TA+PRE | 369.41 |
|  | TA+PRE+PHO+RH | 345.62 |  | TA+PRE+PHO | 375.72 |
